# Healed Varicella Pneumonia: A Case of Diffuse Pulmonary Microcalcifications

**DOI:** 10.7759/cureus.15890

**Published:** 2021-06-24

**Authors:** Amrutha Mylarapu, Varun Yarabarla, Rebekah M Padilla, Madeline Fasen, Pramod Reddy

**Affiliations:** 1 Radiology, Philadelphia College of Osteopathic Medicine, Atlanta, USA; 2 Neurology, Philadelphia College of Osteopathic Medicine, Atlanta, USA; 3 Diagnostic Radiology, University of Florida College of Medicine – Jacksonville, Jacksonville, USA; 4 Internal Medicine, University of Florida College of Medicine – Jacksonville, Jacksonville, USA

**Keywords:** varicella-zoster, varicella pneumonia, pulmonary microcalcifications, chicken pox, ground-glass opacities, lung calcifications, high-resolution ct scan, plain radiography, chest x-ray (cx-ray), pulmonary tuberculosis

## Abstract

Varicella pneumonia is a potentially fatal complication of the Varicella-zoster virus (VZV), which causes the well-known chickenpox disease of childhood. Identifying this type of pneumonia by characteristic features is important for radiologists and radiology residents. Typical manifestations of active Varicella pneumonia include diffuse pulmonary nodules, which may mimic other diseases. Healed Varicella pneumonia can present as diffuse, calcified pulmonary micronodules. We describe a case of healed Varicella pneumonia in a patient with a history of remote VZV infection.

## Introduction

Varicella-zoster virus (VZV) is primarily known for causing childhood chickenpox, a highly contagious disease that affects many body systems including the skin, central nervous system, and lungs. Primary chickenpox affects the skin and is generally self-limiting, but Varicella pneumonia is a potentially fatal complication associated with the disease, which may mimic other diseases [1]. The development of multiple, diffuse pulmonary nodular calcifications with noncalcified lymph nodes is uncommon, with an incidence of 1.0 to 2.3 cases per 400 VZV cases [2]. Other studies have shown that mild pneumonitis is present in about 16% of radiographic cases [2].

The risk of primary Varicella pneumonia is more prevalent in adults, with an increased risk if the patients are pregnant, have chronic lung disease, or are immunocompromised [3,4]. Smoking also increases a patient’s susceptibility to Varicella pneumonia, as smoking leads to decreased activation of the alveolar macrophages [3]. An increased number of skin lesions from primary VZV infection showed a correlation with an increased risk of developing Varicella pneumonia [4]. Contact with chickenpox, especially a history of contact with one’s own child, also showed a high chance of developing Varicella pneumonia [4]. It was also shown that patients with previous contact with Varicella have an increased risk of morbidity and mortality, and the overall mortality rate from Varicella pneumonia in recent years has been reported to be around 6%, which is significantly decreased from 19% in the late 1960s [4]. The introduction of the Varicella vaccine has subsequently led to more decline in Varicella incidence and mortality [5]. By the early 2000s, data show that Varicella associated deaths, including deaths from Varicella pneumonia of adults older than 50 years, dropped by 16% [6].

As Varicella pneumonia is a complication from VZV, patients generally present with a primary Varicella infection and then develop associated chest tightness, tachypnea, cough, dyspnea, fever, pleuritic chest pain, and hemoptysis [4]. In some cases, symptoms of chest discomfort can begin before the onset of the rash. Primary Varicella infection spreads via droplet transfer and the rate of infectivity increases drastically in areas of close proximity [5].

The diagnosis of Varicella pneumonia is based on presenting characteristics and an increase in VZV titers [7]. Varicella pneumonia may present with multiple lung nodules on computed tomography (CT), which can present similarly to a variety of other diseases. Kim et al. described the nodules of Varicella pneumonia on a high-resolution CT scan as a nodule of 1-10 mm in diameter with a distinguished shadow surrounding the node [8]. Although these findings are not immediately diagnostic of Varicella pneumonia, they allow for a list of differential diagnosis which includes nodular amyloidosis, calcified metastasis, hyalinizing granuloma, epithelioid hemangioendothelioma, necrobiotic nodules of rheumatoid arthritis, multiple pulmonary chondromas, and progressive massive fibrosis [1].

## Case presentation

A 57-year-old female with a past medical history of hypertension, intravenous (IV) amphetamine and cocaine use presented to the emergency department for left upper extremity pain, swelling, and lethargy. The patient admitted to IV drug use two days prior in the symptomatic left forearm. A review of systems was positive only for shortness of breath and chest pain. History revealed a recent admission at an outside hospital for methicillin-resistant Staphylococcus aureus (MRSA) bacteremia treated with IV antibiotics followed by a long oral antibiotic course. She denied a history of endocarditis, sick contacts, or recent travel. She denied a recent rash. There was no history of tuberculosis (TB), HIV, Hepatitis B or C, sarcoidosis, or other immunocompromising diseases.

**Figure 1:**
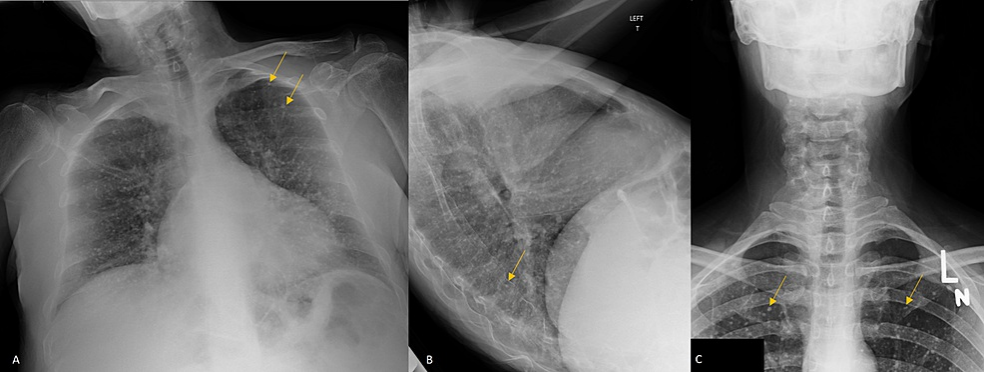
AP (A) and lateral (B) chest radiographs taken on admission demonstrate numerous, subcentimeter calcified pulmonary nodules (yellow arrows). An AP neck radiograph (C) taken approximately 10 years prior to admission demonstrates multiple calcified nodules (yellow arrows) confirming chronicity. AP: anteroposterior

**Figure 2:**
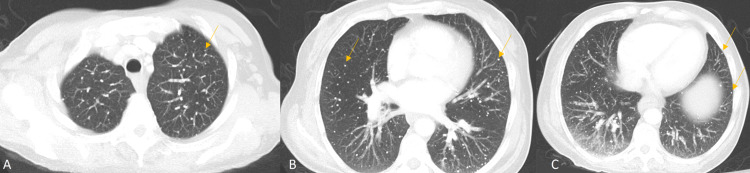
CT images utilizing a lung kernel and maximum intensity projections at apical (A), mid-lung (B), and basilar (C) axial slices. Multiple pulmonary calcified nodules (yellow arrows) are diffusely distributed through the lungs. No ground-glass opacities are present to indicate an acute inflammatory process.

In the emergency room, vitals revealed a mild tachycardia of 101 beats per minute, blood pressure of 133/81 mmHg, respiratory rate of 22 breaths per minute, oxygen saturation of 94% on 2 liters nasal cannula, and an elevated oral temperature of 101.2 degrees Fahrenheit. She was started on IV clindamycin for left extremity cellulitis. Based on her respiratory symptoms and presentation of sepsis, workup initially included a frontal and lateral chest radiograph. The radiographs were significant for diffuse pulmonary nodules (Figures 1A, 1B). CT of the chest was performed (Figures 2A-2C), which showed calcified pulmonary nodules without ground-glass opacity, lymphadenopathy, or other acute findings. The nodules were predominantly bibasilar and measured less than 3 mm. Due to limited history at the time of findings, the differential diagnosis was broad, including miliary TB, granulomatous disease, metastasis, and other diffuse pulmonary diseases. The patient was admitted to the hospital for sepsis and isolated for suspected TB. Physical exam was significant for left antecubital erythema and swelling and tachypneic, shallow respirations with bilateral rales. She had no petechial or maculopapular rash.

After initial lethargy improved, additional history from the patient narrowed the differential diagnosis. She admitted to a history of “chickenpox pneumonia” that occurred 20-30 years prior while she was pregnant. She was unable to recall the treatment at the time of diagnosis but reported her daughter had no complications as a result. She mentioned having “abnormal” chest imaging since that event. Interestingly, a prior neck radiograph from approximately ten years prior was noted to have similar calcified pulmonary nodules (Figure 1C). This narrowed the differential to a chronic, likely healed disease process.

Two positive blood culture results for Streptococcus pyogenes were obtained over the course of her stay. Coronavirus-19 testing was negative and Varicella IgG returned immune. Antibiotics were transitioned from IV clindamycin to IV vancomycin and IV piperacillin/tazobactam. Tachypnea and shortness of breath improved.

Over the course of hospitalization, two sequential negative acid-fast bacilli tests, nucleic acid amplification (NAA) TB test and a purified protein derivative (PPD) test were negative, ruling out TB. HIV tests were negative. The Hepatitis panel was positive for Hepatitis C; however, a quantitative viral load was undetectable. Additionally, echocardiography was normal with an ejection fraction of 65% without valvular vegetation.

During the hospital course, the patient developed opiate withdrawal symptoms and left the hospital against medical advice. However, after discharge, sputum cultures were positive for Klebsiella pneumoniae and blood work revealed positive Varicella IgA.

## Discussion

Varicella pneumonia can be a fatal complication of VZV. Generally, patients present with respiratory distress within one to six days of the onset of a rash [5]. Although radiographic images cannot be used to definitively diagnose Varicella pneumonia, imaging is useful in narrowing the differential diagnosis. Chest radiographs for Varicella pneumonia can show multiple 5-10 mm ill-defined nodules that may be confluent and may come and go in different areas of the lungs [8]. The acute phase can present with hilar adenopathy and small pleural effusions [8]. Small, round consolidations can resolve within one week of the disappearance of the rash or persist for months [8]. In other patients, such as ours, the calcifications can be persistent and present as diffuse, subcentimeter nodular densities with otherwise normal lung findings [8].

Compared to radiographic findings, CT findings of Varicella pneumonia in immunocompetent patients have been described as multiple nodules of soft-tissue densities ranging from 5 to 10 mm in diameter with no calcifications and appearing bilaterally [7-9]. Kim et al. described three patients with Varicella pneumonia, and the CT scan findings from these patients showed nodules with a halo of ground-glass attenuation, patchy ground-glass attenuation, and coalescence of lesions [8]. They also indicated that high-resolution CT was able to more precisely identify Varicella pneumonia than chest radiographs [8]. This was substantiated by the fact that Kim et al. were able to identify 5-10 mm nodules with ground-glass attenuation in the CT scans that were not present in the radiographs [8]. The presence of ground-glass opacities can also be used to characterize acute Varicella pneumonia, while healed Varicella pneumonia does not tend to have this finding of inflammation, as seen in our case (Figures 2A, 2B).

When considering a diagnosis of Varicella pneumonia, some key diagnoses must be ruled out. Presentation of nodular opacities in the lungs can indicate a variety of disease processes ranging from pulmonary metastasis to TB. When comparing the findings of Varicella pneumonia to metastasis, metastasis generally presents as poorly defined nodules with infiltration into the vessels of the chest wall. Metastatic nodules are also larger ranging from 3 to 10 mm, while Varicella tends to present as nodules ranging from 5 to 10 mm [10]. Another differential diagnosis to strongly consider is miliary TB which presents with 1-3 mm nodules that are uniformly distributed in the lung [1,11]. The biggest differentiating factor is that miliary TB will elicit a positive TB test. Occupational diseases such as silicosis and coal worker’s pneumoconiosis also present with small, calcified nodules, a similar presentation to Varicella pneumonia. One strong indication to consider silicosis over Varicella pneumonia is the presence of eggshell calcifications [12]. Coal worker’s pneumoconiosis on the other hand can be distinguished from Varicella pneumonia as it has nodules that will not present on plain film [12]. Table 1 defines certain characteristics of possible differentials when a patient presents with nodular changes in the lung parenchyma [1,10,11,13].

**Table 1:** Differential Diagnosis of Calcified Pulmonary Nodules

Differential Diagnosis	Etiologies and Characteristics	Findings on Imaging
Calcified Granulomata	Healed calcification; Most common finding	Calcifications may be central or diffuse and range from 2 to 5 mm
Healed Varicella	Calcified nodules	Micronodular calcifications ranging from 1 to 3 mm
		Does not present with nodal calcifications
Miliary Tuberculosis	Calcified nodules	1-3 mm nodules, uniform in size and distribution
Calcified Metastasis	Can result from sarcomas (osteosarcoma, chondrosarcoma, synovial sarcoma and giant cell tumor of the bone) or carcinomas (mucin-producing carcinoma, thyroid malignancy, and treated metastatic choriocarcinoma)	Nodules are poorly defined and size ranges from 3 to 10 mm
		Calcification generally occurs in the upper lobes
		Calcifications in the vessels of the chest wall
Nodular Amyloidosis	Excessive deposition of amyloid light chain protein	50% of nodules calcify or ossify
Hyalinizing Granuloma	Can be caused by Histoplasma or Mycobacterium	Multiple fibrosing, well-defined nodules or solitary, slow-growing nodule
Epithelioid Hemangioendothelioma		Multiple bilateral nodules
		Calcification 10-20 years after diagnosis
Necrobiotic Nodules of Rheumatoid Arthritis	Presents with pleural disease, interstitial pneumonia, and necrobiotic nodules	Nodules contain cavitation
Progressive Massive Fibrosis		Bilateral mass-like consolidations
		Parenchymal scarring
Occupational Diseases	Silicosis or Coal Worker’s Pneumoconiosis	Diffuse, calcified small nodules
		CT findings show randomly distributed small well-defined nodules
Pulmonary Hemosiderosis	Idiopathic	Recurrent alveolar hemorrhage
		Centrilobular nodular opacities
Mitral Stenosis	Acquired	Small multifocal calcified nodules
Pulmonary Alveolar Microlithiasis	Genetic	Minute micronodules
		Small subpleural cysts
		Calcification of interlobular septa

IgA antibody can be detected in patients who have recovered from VZV [14]. Elevated IgA in recovered patients may indicate early reactivation of the virus, even when IgG is present from prior infection. Interestingly, our patient had no signs of cutaneous lesions or symptomology even though her IgA levels were elevated. This contrasts with findings from Cradock-Watson et al. who were able to identify rising IgG, IgM, and IgA titers within 2-5 days after the onset of the rash, while titers of IgA and IgM were minimal within six weeks [14]. There were no clinical signs of acute infection in this patient’s case.

Varicella pneumonia can be diagnosed utilizing bronchoalveolar lavage, which checks for the presence of varicella-zoster viral DNA via polymerase chain reaction (PCR) [15]. Previous case reports have used skin biopsies to test for VZV if the patient presented with a vesicular-pustular rash. Skin biopsies may show subepidermal vesicles with multi-nucleated giant cells [16]. Lab work may show low titers of IgA.

Treatment for patients presenting with Varicella pneumonia would be to begin IV acyclovir with intensive supportive care [15,16]. The use of steroids as part of the treatment regimen remains controversial, yet a multitude of case reports have included steroids as part of care management [17,18]. Treatments using Varicella IG (VZIG) have been recorded as treatment or prophylaxis, but in most cases, the use of immunoglobulins is time-sensitive with efficacy decreasing as the disease progresses [17]. Some case reports have indicated the use of VZIG in immunocompromised patients or patients who have disseminated Varicella infections, but no studies have quantified the efficacy of VZIG in the treatment of severe Varicella infections [17].

## Conclusions

Diffuse pulmonary calcifications found on imaging raise concern for a plethora of differential diagnoses. The specific etiology must be narrowed by a clinical history, physical exam, and appropriate laboratory testing while prior imaging plays a vital role in determining chronicity and potentially eliminating acute etiologies. Chronic calcified pulmonary nodules should raise concern for a healed pulmonary disease such as Varicella pneumonia.
